# G-protein coupled estrogen receptor 1 *(GPER1)* expression improves survival of glioblastoma patients depending on sex and age: biological mechanisms and epigenetic regulation

**DOI:** 10.3389/fendo.2026.1923928

**Published:** 2026-09-25

**Authors:** Alice Gantner, Laetitia Soudre-Bau, Yann Bailly, Alicia Chateau, Fabien Rech, Hélène Dubois-Pot-Schneider, Hélène Dumond

**Affiliations:** 1 Université de Lorraine, CNRS, CRAN, Nancy, France; 2 Université de Lorraine, CHRU-Nancy, Service de Neurochirurgie, Nancy, France

**Keywords:** database mining, epigenetic regulation, glioblastoma, GPER, prognosis, sex-specific, transcriptomic profile

## Abstract

**Introduction:**

In 2021, WHO classification of brain tumors adopted an integrated approach, combining histopathological features with key molecular alterations, notably redefining astrocytoma and glioblastoma entities. Among gliomas, glioblastoma has the worst prognosis, with a median patient survival rate of no more than 15 months. To date, no treatment is effective and there is no targeted therapy available. Recent research underscores the influence of sex and sex hormones in gliomagenesis, development and response to treatment. Indeed, glioblastoma exhibit sex-based disparities in adults, with a higher incidence of these tumors and shorter survival in males compared to females. Sex differences tend to narrow in older populations, suggesting that beside genetic sex or acquired gender-biased specificities, sex hormones play a significant role in this difference. Among the key hormone receptors potentially involved, we focused on GPER, the membrane g-coupled estrogen receptor.

**Methods:**

Expression, methylation and clinical data from TCGA-LGG and TCGA-GBM datasets were reclassified according to WHO 2021. *GPER1* expression level was correlated to patient overall survival and methylation status of the *GPER1* locus. Underlying mechanisms were unraveled *in vitro* by transcriptomic and functional analyses of GPER-overexpressing cells.

**Results:**

A high expression of *GPER1* predicted overall survival depending on age and sex, especially for females aged less than 55 years. Functional analysis of *GPER1*-correlated genes indicated that *GPER1* high expression is associated with cell cycle impairment. *In vitro*, GPER protein overexpression impaired U251 cell proliferation, which confirmed the benefice of *GPER1* high expression against glioblastoma. Since methylation of *GPER1* locus was previously shown to control its expression in other cancers, we finally identified 3 CpG sites whose methylation level were inversely correlated with *GPER1* expression. Interestingly, the combined low methylation level of these 3 CpG sites is associated with prolonged overall survival in females under the age of 55, suggesting that methylation at these loci may contribute to *GPER1* expression control in glioblastoma.

**Conclusion:**

The present findings identify a potential mechanism of *GPER1* regulation in glioblastoma and suggest that GPER could be a therapeutic target of interest, notably for non-menopaused women.

## 1 Introduction

Gliomas are among the most common primary tumors of the central nervous system (CNS) in adults, and their classification is regularly updated. The latest WHO classification from 2021 adopted an integrated diagnostic approach, combining histopathological features with molecular profiling. According to this classification, glioma are stratified into four grades and glioblastoma (GB) is designated as a WHO grade 4, IDH-wildtype (IDHwt) diffuse astrocytic glioma (1). With the worst prognosis, GB is the most common tumor of all primary CNS tumors. The age-adjusted incidence rate is 2.74 per 100,000 people, and the median age at diagnosis is 65 years (2). To date, the standard treatment used is the Stupp protocol, which involves surgical resection followed by concomitant chemoradiotherapy based on temozolomide (TMZ) (3). This treatment slightly increases median survival from 9 months to 16 months, without being curative (4). This protocol is applied to all patients, but recent research underscores the influence of sex and sex hormones on gliomagenesis, development and response to treatment. Indeed, the incidence is higher in men than in women with the sex ratio 1.6:1 and women have a better survival rate following the Stupp protocol than men (25.5 and 15 months respectively) (5). This gender difference tends to decrease in older populations, suggesting a potentially protective role for female sex hormones (6). This was also confirmed in a orthotopic xenograft rat model where model tumor growth was reduced in females compared to males or ovariectomized females (7).

Estrogens can bind to nuclear estrogen receptors α (ERα) and β (ERβ) or G protein-coupled estrogen receptor (GPER), a 7 transmembrane domain protein that belongs to class A (rhodopsin-like) GPCR family (8, 9). GPER, encoded by *GPER1* gene, is localized in the plasma membrane when N-terminal glycosylated and correctly folded and/or in the endoplasmic reticulum membrane (10). Following agonist binding, it activates heterotrimeric G proteins which initiates rapid non-genomic signaling through several pathways such as calcium mobilization, cAMP synthesis and kinase pathway activation (11, 12). To date, limited data is available regarding the prognostic value of these receptors in GB as defined by the 2021 WHO classification. From a supervised *in silico* analysis of TCGA-LGG (Low Grade Glioma) and TCGA-GBM (Glioblastoma) datasets, based on the 2016 WHO classification we showed that low *ESR1* expression, that encodes ERα, is associated to a better overall survival in IDHwt grade 3 tumor (13). However, *ESR1* encodes several ERα isoforms that exhibit anti- or pro-proliferative effects (8). High level of ERα66 canonical protein (66 kDa) was associated with longer survival in GB patients in both sexes (14). Conversely, the 36 kDa ERα isoform (ERα36)/EGFR downregulation disturbed growth and invasion *in vitro* (15). In the study of Hirtz et al. (13), *ESR2* expression, that encodes at least 5 ERβ isoforms, showed no significant association with clinical outcome although ERβ1 and ERβ5 were shown to display anti- or pro-tumoral properties, respectively (16). In contrast, high *GPER1* expression is of better survival prognosis in either grade 3 or grade 4 IDHwt tumors, especially for female patients treated with Stupp protocol (13, 17). This finding suggests that the expression of *GPER1* should be coupled with the activation of the receptor by female hormones to extend survival. Indeed, *in vitro* functional assays and *in vivo* xenograft experiments indicated that the GPER agonist G-1 triggered anti-proliferative effects on GB cells (17, 18). Another study performed by Gutiérrez-Almeida et al. indicated an opposite effect since a treatment with G-1 or the GPER antagonist G-15 resulted in an increase or a decrease of cell proliferation in GB, respectively (19). Nevertheless, many publications questioned the agonist activity of G-1 and even demonstrated that GPER may trigger ligand-independent signaling (20, 21). Taken together, those data underscore the importance of understanding the molecular mechanisms underlying the effects of a high *GPER1* expression depending on patients’ sex and hormonal status.

In this study, we developed an original workflow to select GB patients as defined by WHO 2021 and reclassified by Mendonça et al. (22). The analysis of clinical and transcriptomic data demonstrated that a high level of *GPER1* expression is associated with enhanced survival outcomes in female patients under the age of 55 (u55 in the text). An enrichment analysis revealed a negative correlation between *GPER1* expression and cell cycle control genes, specifically within this group. These findings were corroborated by a GPER overexpression experiment in GB cells, which supports the hypothesis that GPER induction can block tumor cell proliferation and enhance survival. Since methylation of *GPER1* locus was previously shown to control its expression in other cancers, we finally identified key CpG sites that may impact the epigenetic regulation of *GPER1* expression in GB.

## 2 Materials and methods

### 2.1 Data collection of TCGA data and pre-processing

The data provided by GDC data portal were acquired using Bioconductor R package TCGAbiolinks in R statistical software (retrieved from https://www.R-project.org/; v 4.5.1) as proposed by Silva et al. (23). Two projects from The Cancer Genome Atlas (TCGA) Research Network (https://www.cancer.gov/tcga, accessed on July 2025) were selected: TCGA-LGG and TCGA-GBM. Three types of data were downloaded for this study: DNA Methylation Data, RNA sequencing data and Clinical data. Based on clinical data, only unique samples (unique and most represented center codes) from patients with primary tumors, identified as IDHwt, grade 3 (G3) or grade 4 (G4) (according to WHO 2016) (24, 25), overall survival data and sex information provided were conserved. The 308 patients selected were stratified by sex and age, under or over 55 years old (u55 or o55) (**Figure 1**). The baseline patient characteristics table is presented in **Supplementary Table 1**.

**Figure 1:**
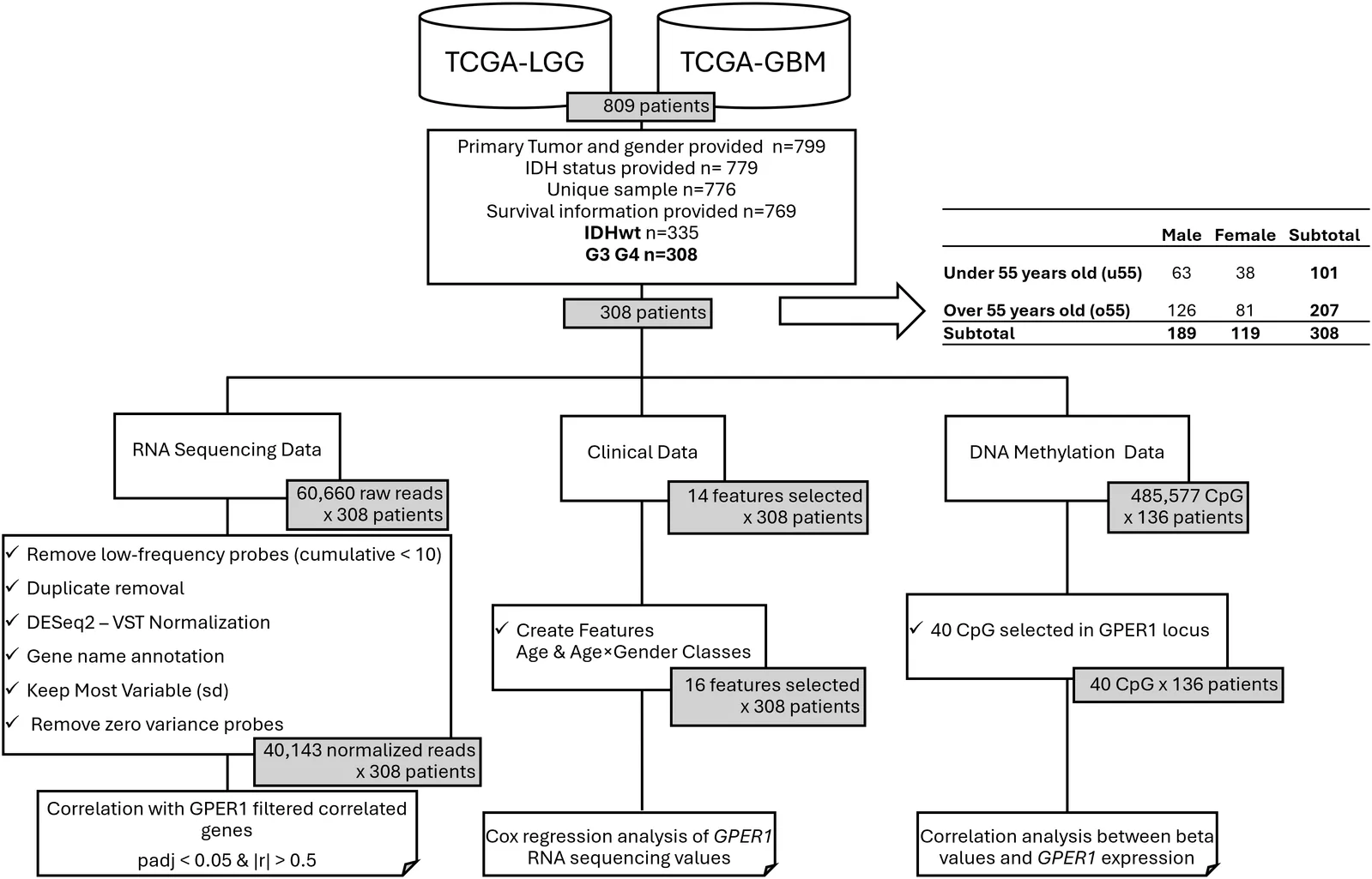
Schematic representation of the study workflow using TCGA datasets.

### 2.2 Survival analysis of TCGA data

A univariate analysis was used to test the link between patient survival and patient sex and age. After performing a first survival analysis on the 4 groups of patients by age and sex, patients were subdivided into 2 groups of high or low *GPER1* expression (8 groups total). The optimal cut-point (surv_cutpoint function, R package survminer) was calculated with the maximally selected rank statistics (cutpoint = 9.2376) and a required minimum proportion of 25% observations per group. Log-rank test was used to compare the Kaplan-Meier survival curves between the 4 or 8 groups, respectively.

### 2.3 Transcriptomic data of TCGA data

Low-frequency probes (cumulative < 10) and duplicate raw reads were removed. Normalization process and variance stabilizing transformation methods (DESeq2-VST) were applied to RNA sequencing data using the R package DESeq2 (26). From the 40,143 normalized reads obtained for the cohort, Spearman correlation was used to identify genes with expression that was correlated to *GPER1* stratified by age and sex (**Figure 1**). Genes with absolute rho | r | > 0.5 and adjusted p value padj < 0.05 were selected.

### 2.4 Methylation data of TCGA data

DNA methylation dataset contained 485,577 CpG sites downloaded on Illumina Human Methylation 450 platform (27). Methylation data were retrieved only for 136 patients (n = 21 for u55 female, n = 20 for u55 male, n = 41 for o55 female and n = 54 for o55 male, respectively) among 308 patients and merged with clinical and RNA sequencing data. The 40 CpG sites within the *GPER1* locus for which methylation data were available for all patients were selected for analysis. For each CpG site, the methylation level (beta value) was correlated to *GPER1* expression using Spearman correlation in the 136-patient cohort, then stratified by age and sex.

### 2.5 Cell culture

U251 cell line was purchased from Cytion (catalog number 300385, Cytion GmbH, Eppelheim, Germany) and maintained in Dulbecco’s modified eagle medium (DMEM) phenol red-free (Gibco) supplemented with 10% fetal bovine serum (FBS), 1% essential amino acids (Sigma-Aldrich), 0.5% non-essential amino acids (Sigma-Aldrich), 0.4% vitamins solution (Sigma-Aldrich), 1.25% sodium pyruvate (SigmaAldrich) and 1% streptomycin/penicillin (Sigma-Aldrich) at 37 °C in a humidified 5% CO_2_ atmosphere.

### 2.6 Transfection

U251 cells were transiently transfected using Lipofectamine 3000 transfection reagent (Invitrogen, L3000-008) according to the manufacturer’s instructions. Cells were plated at a density of 2.10^5^ cells per well in 6-well plates in the standard growth medium. After 24 h, cells were transfected with 1 μg plasmid of GPER-Tango (Addgene plasmid # 66299; http://n2t.net/addgene:66299; RRID: Addgene_66299) (28) (GPER+) or with 1 µg plasmid of pcDNA3.1 (control) as control. The growth medium was renewed 6 hours after transfection.

### 2.7 *In vitro* transcriptomic analysis

Microarray experiment was performed on mRNAs from 4 independent biological replicates of control or GPER+ U251 cells. Microarray gene expression profiling was conducted using the low-input QuickAmp labeling kit and human SurePrint G3 8 × 60 K pangenomic microarrays (Agilent Technologies, Santa Clara, CA, USA). Gene expression data were processed using Feature Extraction (Agilent Technologies). The data discussed in this publication have been deposited in NCBI’s Gene Expression Omnibus (GEO) and are accessible through GEO Series accession number GSE332587 (https://www.ncbi.nlm.nih.gov/geo/query/acc.cgi?acc=GSE332587). The following secure token has been created to allow review of record GSE332587 while it remains in private status: ibszumagvpwfteb. Raw data were processed and analyzed using GeneSpring version 15.5 software (Agilent Technology, Santa Clara, CA, USA). Raw microarray signal intensities were normalized across all samples using the 75th percentile normalization method. Differentially expressed genes were identified by a t-test with a p value p < 0.05 and an absolute fold change |FC| > 1.7. Clustering analysis was performed using Cluster 3.0 and TreeView 1.1.6r4 (Java Treeview) using uncentered correlation and complete linkage.

### 2.8 Enrichment analysis

Gene set enrichment analysis (GSEA, Gene Set Enrichment Analysis) was used to check whether an *a priori* defined set of genes shows statistically significant, concordant differences between two biological states. Unsupervised GSEA was performed with Gene Ontology (GO) in the C5 collection of ontology gene set from the molecular signatures database (MSigDB) (29, 30). A Venn diagram was used to visualize the intersection of GPER-correlated genes across patient groups (31).

### 2.9 Connectivity map

The clue.io platform (https://clue.io/query) was used to compare the GPER overexpression signature to the L1000 gene-expression profiles obtained from the treatment of a variety of cell types with reference perturbagens, available in the public LINCS Connectivity Map (CMap) ressource (32). The CMap connectivity score (tau) was used to identify these perturbagens with a selection threshold of tau > 90.

### 2.10 Flow cytometry

Seventy-two hour after transfection, control or GPER+ U251 cells were harvested, the cell pellets were resuspended in phosphate-buffer saline and fixed overnight in 70% cold ethanol at 4 °C. Cells were then stained with propidium iodide in the presence of RNase for 30 min. Cell cycle distribution was analyzed by measuring DNA content using the Celesta Sorp flow cytometer (BD Biosciences) and data were analyzed with FlowJo v11.2 Software (BD Life Sciences).

### 2.11 Western blot

Briefly, the cells were collected and lysed in RIPA Buffer containing protease and phosphatase inhibitors. After addition of 4X Laemmli (v/v), total proteins were separated by 12% polyacrylamide gels prepared in accordance with the manufacturer’s instructions TGX Stainfree FastCast Acrylamide kit (Biorad 1610185). Proteins were then transferred using Trans-Blot Turbo Mini 0,2 µm Nitrocellulose Transfer (Biorad 1704158) and a transfer cassette Trans-Blot TurboTM Transfer System (Biorad 1704150). After transfer, total lane proteins were visualized using the ChemiDoc™ (Biorad, France) and used for normalization. Non-specific binding sites were blocked in TBST buffer (60 mM Tris–HCl, 150 mM NaCl, 0.1% Tween 20) containing 3% bovine serum albumin and incubated overnight at 4 °C with anti-GPER polyclonal goat antibody (1:2000, R&D systems, AF5534) and anti-EGR1 polyclonal rabbit antibody (1:1000, SantaCruz, sc-189). Next, the membranes were washed 4 times with TBST and incubated for 1 h with a horseradish peroxidase-coupled anti-goat antibody (1:10000, Azure Biosystems, AC2149) and a horseradish peroxidase-coupled anti-rabbit antibody (1:10000, Cell Signaling, 7074S). Luminescence was detected after addition of ECL prime western blotting (GE Healthcare RPN2236) with ChemiDoc™ (Biorad, France).

### 2.12 RT-qPCR

Total RNA was extracted from GB cells or GB patient samples using the E.Z.N.A HP total RNA kit (Omega Biotek, Norcross, GA, USA), following the manufacturer’s instructions. Reverse transcription of 500 ng total RNA was performed using the iScript Reverse Transcription Supermix for RT-qPCR kit (BioRad, Hercules, CA, USA). RT-qPCR were performed using iTaq Universal SYBR Green Supermix (BioRad, Hercules, CA, USA) with CFX96 Real Time PCR Detection System and software (Biorad, Hercules, CA, USA). The primers used are: *EGR1* forward 5’-AGCAGCACCTTCAACCCTCAGG-3’ and reverse 5-GAGTGGTTTGGCTGGGGTAACT-3’; *GPER1* forward 5’- TTCCGCGAGAAGATGACCATCC-3’ and reverse 5’-TAGTACCGCTCGTGCAGGTTGA-3’; and *U6* forward 5’- CGGGTTTGTTTTGCATTTCT-3’ and reverse 5’- AGTCCCAGCATGAACAGCTT-3’. Assays were performed at least in triplicate, and the mean values were used to calculate the expression levels using the Starting Quantity (SQ) method, referring to the *U6* housekeeping gene expression.

### 2.13 GB tumor from patients

Six female IDHwt GB patient samples were collected from the neurosurgery department of the CHRU in Nancy. All patients gave informed consent (Collection reference n°DC-2019-3739).

### 2.14 Statistical analysis

*In vitro* data were analyzed using GraphPad Prism 8 (https://www.graphpad.com). Normal distributions of data were evaluated by Shapiro-Wilk test. Statistical significance was evaluated by Students’s t-test for two-by-two comparison for unpaired data. Results are expressed as means ± SD (standard deviation). The number of independent experiments performed is indicated as n in each figure legend.

## 3 Results

### 3.1 *GPER1* expression is of survival prognostic value for GB u55 female patients

As a preliminary step prior to investigating the role of *GPER1* expression in GB, the clinical, transcriptomic and methylation data available in the TCGA-GBM and TCGA-LGG datasets were reclassified in accordance with the 2021 WHO classification. From these two datasets, we selected IDHwt samples (335 patients) and then restricted our population to tumors classified as grade 3 and grade 4 in both original datasets (308 patients; **Figure 1**). Several retrospective studies have shown that GB patients’ sex and age impact their survival (33–35), with a better prognosis among women of childbearing age, suggesting a protective effect of estrogen. Patients were therefore stratified by sex and age. The mean age at natural menopause worldwide is approximately 49 years, although substantial geographical variation exists. Large systematic reviews have reported mean ages ranging from 46 to 52 years across populations, with extreme reported averages varying from 44.6 to 54.5 years depending on geographic, socioeconomic, and lifestyle factors (36, 37). Since TCGA database did not collect data on menopausal status, hormone replacement therapy, hormonal contraceptive use, or circulating sex hormone levels, the hormonal exposure/circulating levels were not available for individual patients. Therefore, the age of 55 years old was selected as a conservative upper-bound value, to differentiate the population based on hormonal status, particularly in terms of estrogen expression levels for females, thus defining 2 groups named under 55 years old (u55) or over 55 years old (o55) for each sex. A significant overall survival (OS) advantage was observed in u55 females (median OS 648 days *versus* 380 days for o55 females, 466 days for u55 males and 388 days for o55 males, respectively; **Figure 2A**).

**Figure 2:**
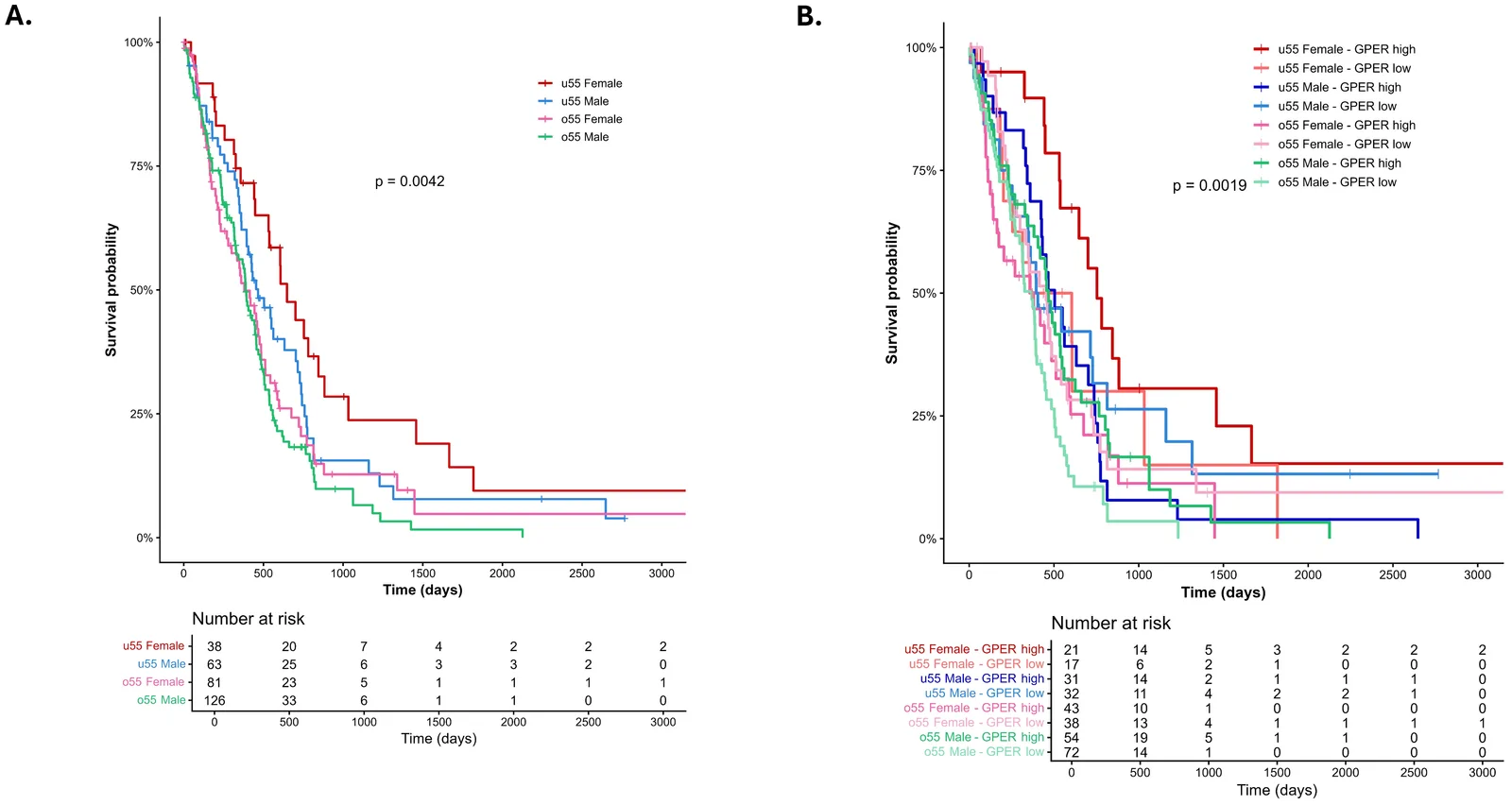
A high expression of *GPER1* is of good prognosis in u55 females. The Kaplan-Meier plots were used to visualize the survival probability for the patients diagnosed with a GB (n = 308). **(A)** Kaplan-Meier estimates according to patient sex and age and **(B)** then adjusted to *GPER1* expression level.

Since *GPER1* was previously shown to be a key prognostic factor in female patients who underwent radiochemotherapy treatment, patients were secondarily stratified by its high *versus* low expression level (17). Although *GPER1* expression was not significantly different between the 4 groups of patients, u55 females which tumor expressed high level of *GPER1* displayed a higher overall survival (754 days) representing at least a 50% to 104% increase compared with the other groups (**Figure 2B**; **Supplementary Figure 1**). This confirmed the key role of GPER in mediating the protective role of estrogens produced in u55 females.

### 3.2 *GPER1* high expression correlates with cell cycle control and mitotic genes in u55 females

In a second step, we searched for *GPER1* correlated genes (absolute rho | r | > 0.5 and padj < 0.05) in each patient group and performed a GSEA signature enrichment to unravel the underlying mechanisms of GPER-dependent better survival in u55 females (**Supplementary Table 2**). As shown in **Figure 3A**, most genes were positively correlated to *GPER1* expression, except for the u55 female group with 81 genes negatively correlated (*versus* 15 genes for the o55 females and 2 genes for u55 or o55 males). Six positively correlated genes (*ESAM*, *FLT4*, *LAMC3*, *ROBO4*, *SLC7A5* and *TMEM204*) were shared between the 4 groups. A group-specific correlation signature of 5 genes (1 negatively and 4 positively correlated to *GPER1*) in o55 males and 25 genes (11 negatively and 14 positively correlated to *GPER1*) in o55 females was obtained but no GO enrichment was identified (**Figure 3B**; **Table 1**; **Supplementary Table 3**). In contrast, a 32 gene specific signature (30 positively and 2 negatively correlated genes to *GPER1*) enriched in biological processes such as “CIRCULATORY_SYSTEM_DEVELOPMENT”, “TUBE_DEVELOPMENT” and “MOLECULAR_TRANSDUCER_ACTIVITY” was identified in u55 males. Moreover, this signature also resembled those of brain endothelial cells (GSEA C8 cell type signature, data not shown) and “BOQUEST_STEM_CELL_DN”. These findings raise the possibility that *GPER1* expression may contribute to the regulation of endothelial-like or vascular programs in u55 males.

**Figure 3:**
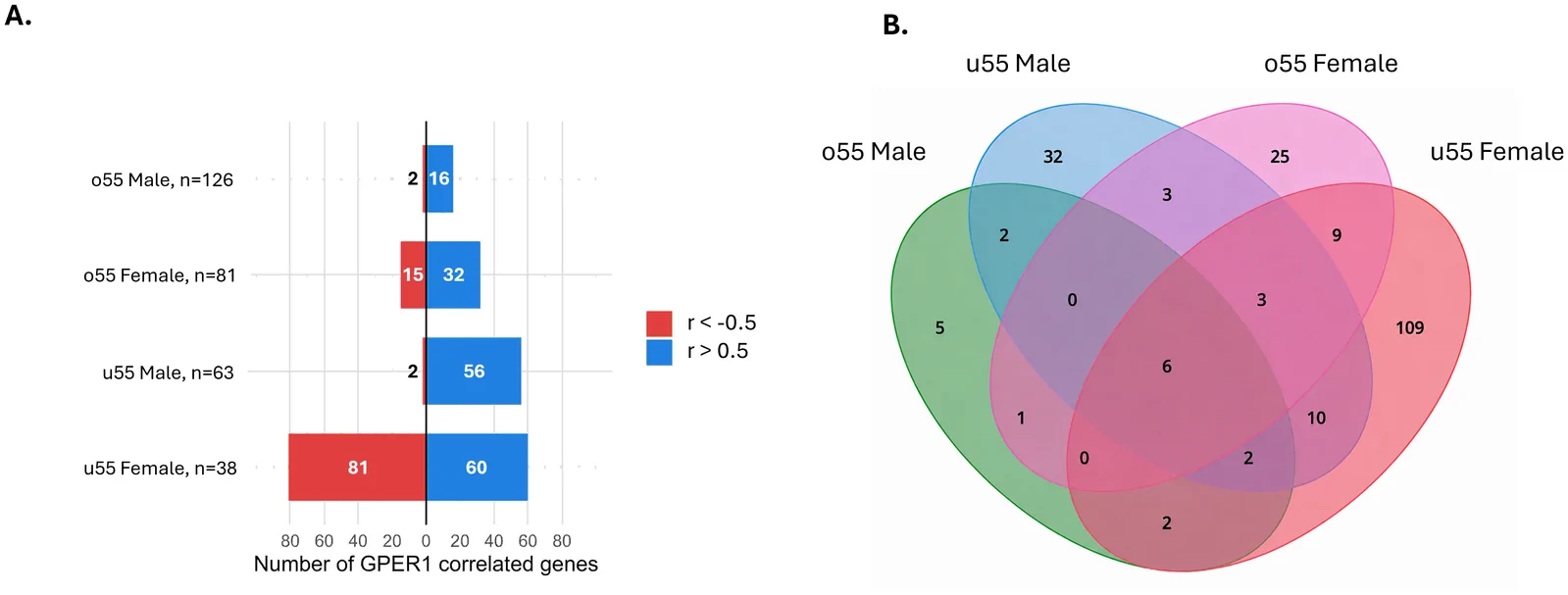
*GPER1* correlated genes. **(A)** Distribution of genes strongly correlated with *GPER1* expression according to patient sex and age. **(B)** A Venn diagram indicating the number of genes found across four groups. The specific signature of each group was defined by the *GPER1* correlated genes identified within it.

**Table 1:** GSEA specific signature enrichment of *GPER1* correlated genes is mainly related to cell cycle in u55 females.

Patient group	Attribute ID	Attribute name	Genes
o55 Males (1 neg and 4 pos genes)	-	-	PLXDC1, AGRN, DOCK6, DAPK3, BBIP1
o55 Females (11 neg and 14 pos genes)	-	-	TBX2, JAG2, PGAP6, GJA4, ESRRA, FOXS1, GRIN2D, STK25, HRC, PPP1R37, CORO1B, PTP4A3, SOX18, ZNF503, DENND1B, RLIM, DNAI4, S100PBP, ZZZ3, AC026341.1, THOC2, C18orf25, PIK3C3, NSUN3, CEP70
u55 Males (2 neg and 30 pos genes)	72359	CIRCULATORY_SYSTEM_DEVELOPMENT	TBXA2R, ACVRL1, ANGPT2, MMRN2, HSPA12B, CD34, HEYL, MIXL1, FOXL1, ARHGEF15, YSLTR2
	60089	MOLECULAR_TRANSDUCER_ACTIVITY	TBXA2R, ACVRL1, ANGPT2, MMRN2, HSPA12B, CD34, HEYL, MIXL1, FOXL1, ARHGEF15, CYSLTR2, TNFRSF4, NMUR1, HCAR1, ADGRL4, FCMR, PEAR1, LRRC32
	35295	TUBE_DEVELOPMENT	ARHGEF15, CYSLTR2, TNFRSF4, NMUR1, HCAR1, ADGRL4, FCMR, PEAR1, LRRC32
u55 Females (33 pos genes)	0010273 0061687 0071294 0071276 0071280 0006882	DETOXIFICATION_OF_COPPER_ION DETOXIFICATION_OF_INORGANIC_COMPOUND CELLULAR_RESPONSE_TO_ZINC_ION CELLULAR_RESPONSE_TO_CADMIUM_ION CELLULAR_RESPONSE_TO_COPPER_ION INTRACELLULAR_ZINC_ION_HOMEOSTASIS	MT3, MT1X, MT1M
	5975	CARBOHYDRATE_METABOLIC_PROCESS	SLC3A2, GYS1, B4GALT7, CHST12, C1QTNF1, GNPTG
u55 Females (76 neg genes)	0010564 0051726	REGULATION_OF_CELL_CYCLE_PROCESS REGULATION_OF_CELL_CYCLE	SMC5, SMC6, SMC4, ETAA1, PRPF40A, XPO1, MRE11, MSH2, SMC2, CCDC66, PKN2, KIF20B, CCP110, APPL1
	0022402 0007049	CELL_CYCLE_PROCESS CELL_CYCLE	SMC5, SMC6, SMC4, ETAA1, PRPF40A, XPO1, MRE11, MSH2, SMC2, CCDC66, PKN2, KIF20B, CCP110, APPL1, PIAS1, ERCC4, SMURF2, CWC22, ZNF638, NOP58, SP3, MSH6
	6312	MITOTIC_RECOMBINATION	SMC5, SMC6, SMC4, MRE11, MSH2, ERCC4
	0051276	CHROMOSOME_ORGANIZATION	SMC5, SMC6, SMC4, MRE11, MSH2, SMC2, CCDC66, ERCC4, MSH6, RIF1, MIS18BP1

According to patient sex and age, GO enrichment of specific *GPER1* correlated genes was performed: top significant GO terms associated with biological processes and genes associated with each enrichment GO term are listed. p < 1.10^-8^.

In the u55 female group, 3 metallothionein encoding genes were positively correlated genes (*MT3*, *MT1X*, *MT1M*) and grouped as metal ion homeostasis and detoxification signature (**Table 1**). Six other positively correlated genes were involved in “GOBP_CARBOHYDRATE_METABOLIC_PROCESS”, underlying the role of GPER in the control of energy metabolism as previously described in other models (38).

As a high level of *GPER1* expression was shown to be indicative of improved survival in u55 females, the focus was placed on the specificity of the correlated genes list for this group. Indeed, 95% of negatively correlated genes (*versus* 54% of positively correlated ones) were retained into the specific signature (**Figure 3B**, **Table 1**; **Supplementary Table 3**), highlighting their biological significance and suggesting that GPER may trigger negative regulations in specific cellular and hormonal contexts. GO terms associated with this list were of high statistical significance and enriched in “CELL_CYCLE_PROCESS”, “REGULATION_OF_CELL_CYCLE_PROCESS”, “MITOTIC_RECOMBINATION” and “GOBP_CHROMOSOME_ORGANIZATION”. This suggested that GPER might be associated to cell cycle blockage and genetic abnormalities in u55 females.

### 3.3 GPER signature supports its antiproliferative effect

To address the gene expression profile alterations under a high versus low *GPER1* expression in GB, we performed a global gene expression analysis in GPER transiently overexpressing cells (GPER+; **Figure 4a**) compared to control U251. By a t-test, we identified 34 deregulated genes (|FC| > 1.7, p < 0.05) upon GPER protein overexpression (**Figure 4b**; **Supplementary Table 4**). The upper part of the dendrogram included 24 genes whose expressions were upregulated by GPER. Among these, 3 upregulated genes, *EGR1* (Early Growth Response 1), *CREB5* (cAMP response element-binding transcription factor) and *JUN*, encode transcription factors that play key roles in the regulation of gene expression and cellular processes such as proliferation. The differential expression of selected genes was validated by RT-qPCR in U251 GPER+ cells as well as in patient samples stratified by *GPER1* expression (**Supplementary Figure 2**). The lower part of the dendrogram clustered 10 genes whose expressions were downregulated by GPER overexpression (**Figure 4b**; **Supplementary Table 4**). Using a connectivity map approach (CMap), we highlighted 137 genes whose overexpression induced a signature similar to that of GPER (tau > 90). Notably, 4 of them (*GPR146*, *GPR148*, *OPN5* and *VN1R2*) belong to GPCR family (class A or orphan) and one-third encode proteins with nucleic acid binding activity. Consistently, GSEA analysis of these 137 genes revealed a significant enrichment (p < 0.001) of GO terms associated with DNA binding or adenylate cyclase activity (data not shown). Interestingly we also identified compounds with a similar (tau > 90) signature to GPER overexpression (**Table 2**). The top classes of perturbagen included “tubulin and microtubules inhibitors”, “ATPase inhibitor”, “CDK inhibitor”, “HDAC inhibitor” and “Toposiomerase inhibitor”. So, these compounds are mainly associated with the inhibition of cell proliferation through disruption of cell cycle progression, mitosis, transcriptional regulation and DNA replication. These data support the antiproliferative effect of GPER overexpression.

**Figure 4:**
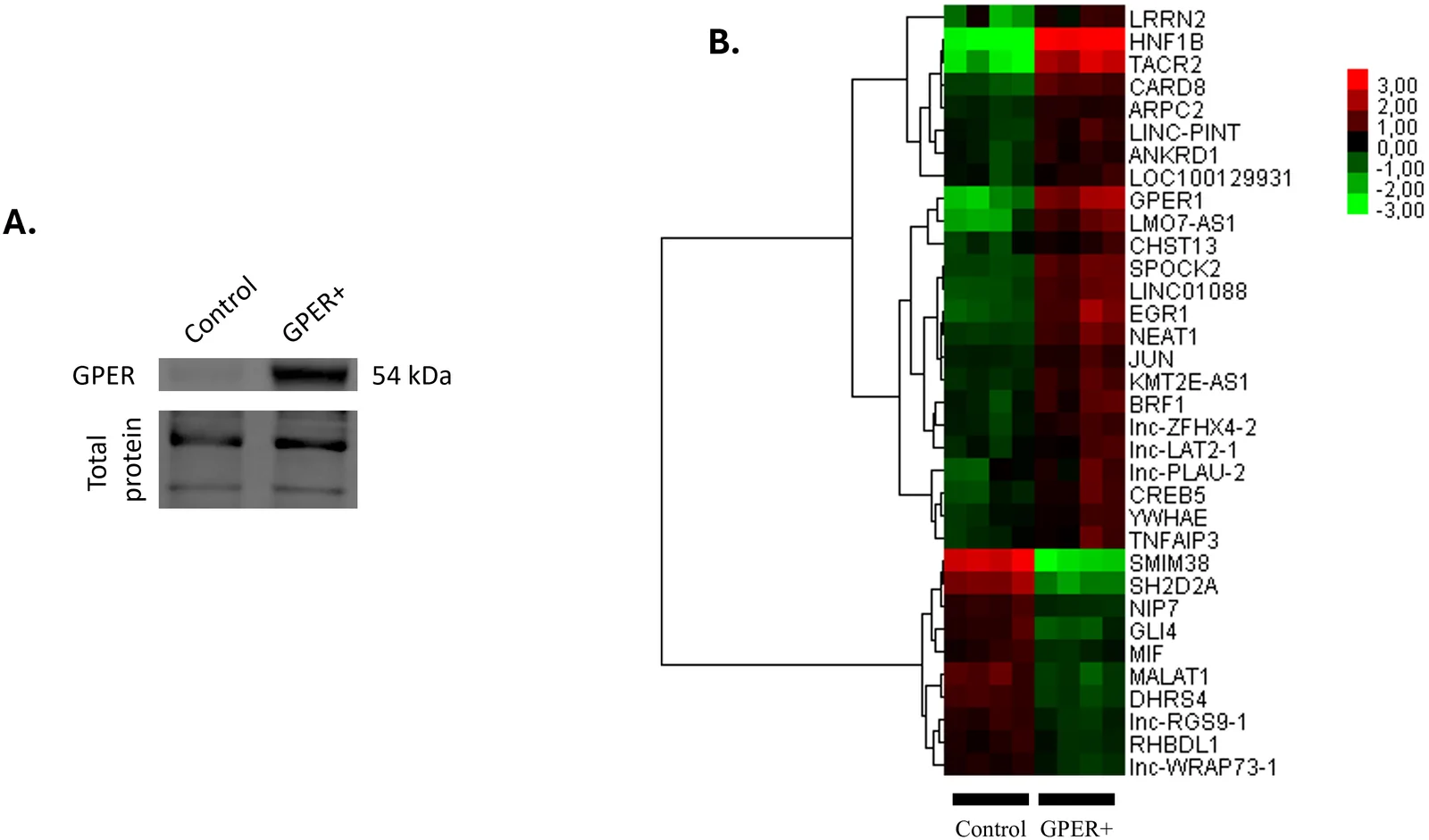
Transcriptomic analysis of GPER+ or control samples. **(A)** Representative image of GPER protein expression in U251 cells detected by Western blotting. n=3. **(B)** Clustering of GPER+ or control samples based on differentially expressed genes (│ FC│ > 1.7, p < 0.05, n=4). The cluster was visualized by Treeview. Green indicates a lower expression and red indicates a higher expression.

**Table 2:** GPER overexpression signature associates to cell cycle disrupting compounds.

Description	Compounds	Median_tau_score
Tubulin inhibitor & Microtubule inhibitor	SA-792574	98.8
	NPI-2358	98.7
	Mebendazole	98.7
	ABT-751	98.52
	Podophyllotoxin	97.85
	Oxibendazole	96.61
	Vinorelbine	96.36
	Vindesine	95.36
	Chelidonine	95.05
	Vinblastine	95
	Flubendazole	94.31
	Nocodazole	94.19
	Vincristine	91.32
ATPase inhibitor	Digitoxigenin	99.19
	Helveticoside	99.19
	Proscillaridin	99.15
	Digitoxin	99.15
	Cinobufagin	99.12
	Cymarin	99.12
	Digoxin	99.12
	Sarmentogenin	99.07
	Ouabain	99.05
	Bufalin	99.01
	Strophanthidin	98.91
	Xanthohumol	98.49
CDK inhibitor	Bisindolylmaleimide-ix	98.45
	Bisindolylmaleimide	98.18
	AT-7519	98.17
	RO-3306	97
	CGP-60474	96.41
	JNJ-7706621	96.34
	PHA-793887	95.99
	Purvalanol-a	94.89
	Alvocidib	93.57
	Kenpaullone	91.21
HDAC inhibitor	Merck60	98.56
	HDAC3-selective	98.2
	Apicidin	97.11
	Phenylbutyrate	95.66
	Panobinostat	95.14
	Entinostat	95.09
	Mocetinostat	93.01
	NSC-3852	92.15
	Dacinostat	91.84
	Tacedinaline	91.38
Topoisomerase inhibitor	SN-38	98.45
	Teniposide	98.03
	Camptothecin	97
	Etoposide	96.3
	Topotecan	96.03
	Amsacrine	95.51
	Irinotecan	94.75
	Pazufloxacin	90.23

Cmap approach allowing the identification of top molecules (tau > 90) whose molecular signature is similar to these of GPER overexpression in U251 cells.

### 3.4 GPER overexpression induced cell cycle impairment in U251 cells

The impact of GPER overexpression on cell cycle progression was further investigated in control and GPER+ U251 cells (**Figure 5**). Flow cytometry analyses revealed that a 72h GPER overexpression increased the number of cells in G2/M by 37.5% but decreased cell population in G0/G1 by 70% and S-phase by 50%, respectively. In addition, GPER overexpression also triggered a 9.4-fold increase of polyploid cells supporting cytokinesis failure. As suggested by the correlation analysis in u55 female patients and the transcriptomic profile of GPER+ GB cells, these results were consistent with a key role of GPER on complete cell cycle control.

**Figure 5:**
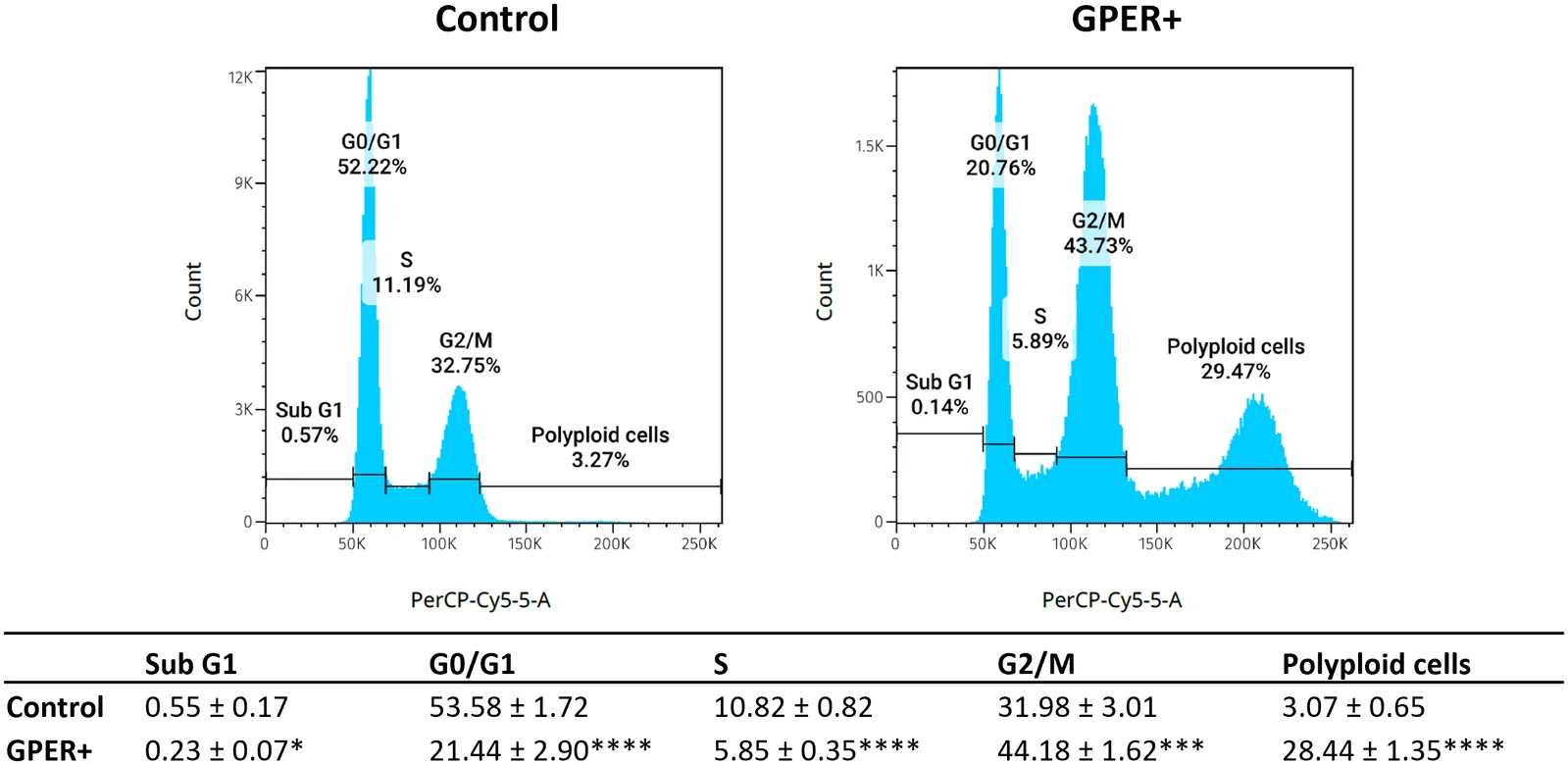
Cell cycle impairment in GPER overexpressing U251 cells. Representative histogram of flow cytometry analyses displaying the distribution of control or GPER+ U251 cells in different phases of the cell cycle. Flow cytometry analysis quantifications are presented as the mean ± SD. n = 4. The level of significance was determined using Student’s t-test with ****indicates p < 0.0001, ***p < 0.001 and *p < 0.05.

### 3.5 A low methylation of key CpG sites in *GPER1* locus is of favorable prognosis in u55 females

Several studies have reported that *GPER1* expression is regulated by epigenetic mechanisms, including promoter methylation, in various cancers. *GPER1* locus contains two CpG islands, located between positions chr7:1,126,579-1,126,910 and chr7:1,131,626-1,133,187 which encompasses 29 and 106 individual CpG sites, respectively (**Figure 6A**; adapted from UCSC Genome Browser) (39). To identify a potential correlation between *GPER1* locus methylation status, *GPER1* expression and patient survival, we analyzed the Illumina 450K methylation data available for 136 IDHwt patients, in both TCGA-LGG and TCGA-GBM datasets (**Figure 1**). The 40 CpG sites located within the *GPER1* locus, for which methylation data were available for all patients in the TCGA dataset, were selected for analysis. We first examined the correlation between the methylation level at each CpG sites and *GPER1* expression (**Figure 6B**). The CpG sites which displayed a significant correlation with *GPER1* expression (p<0.05 and |r|>0.3) and a mean beta value >0.9 were retained for further analysis. Three CpG sites matched those criteria - cg02910650, cg03214053 and cg09510128- were negatively correlated with *GPER1* expression (r = - 0.342, r = - 0.462 and r = - 0.483, respectively). Inverse correlation with *GPER1* expression reached - 0.49 when we summed the beta values of the three key CpG sites (triplet), suggesting that methylation status of these 3 sites may drive *GPER1* expression in GB (**Figure 6C**). This negative correlation of the triplet beta value was found in each sex-and-age patient group (**Supplementary Figure 3**). Then, we explored the survival prognostic value of the CpG triplet methylation. Each patient group was stratified according to high or low methylation level based on the beta value of the CpG triplet. The methylation level of the CpG triplet was cut off in two subgroups (high and low) with the survcutpoint function (cutpoint = 2.8366). Kaplan-Meier estimates indicated that the combined low methylation level of cg02910650, cg03214053 and cg09510128 is associated to prolonged overall survival in u55 females (**Figure 6D**).

**Figure 6:**
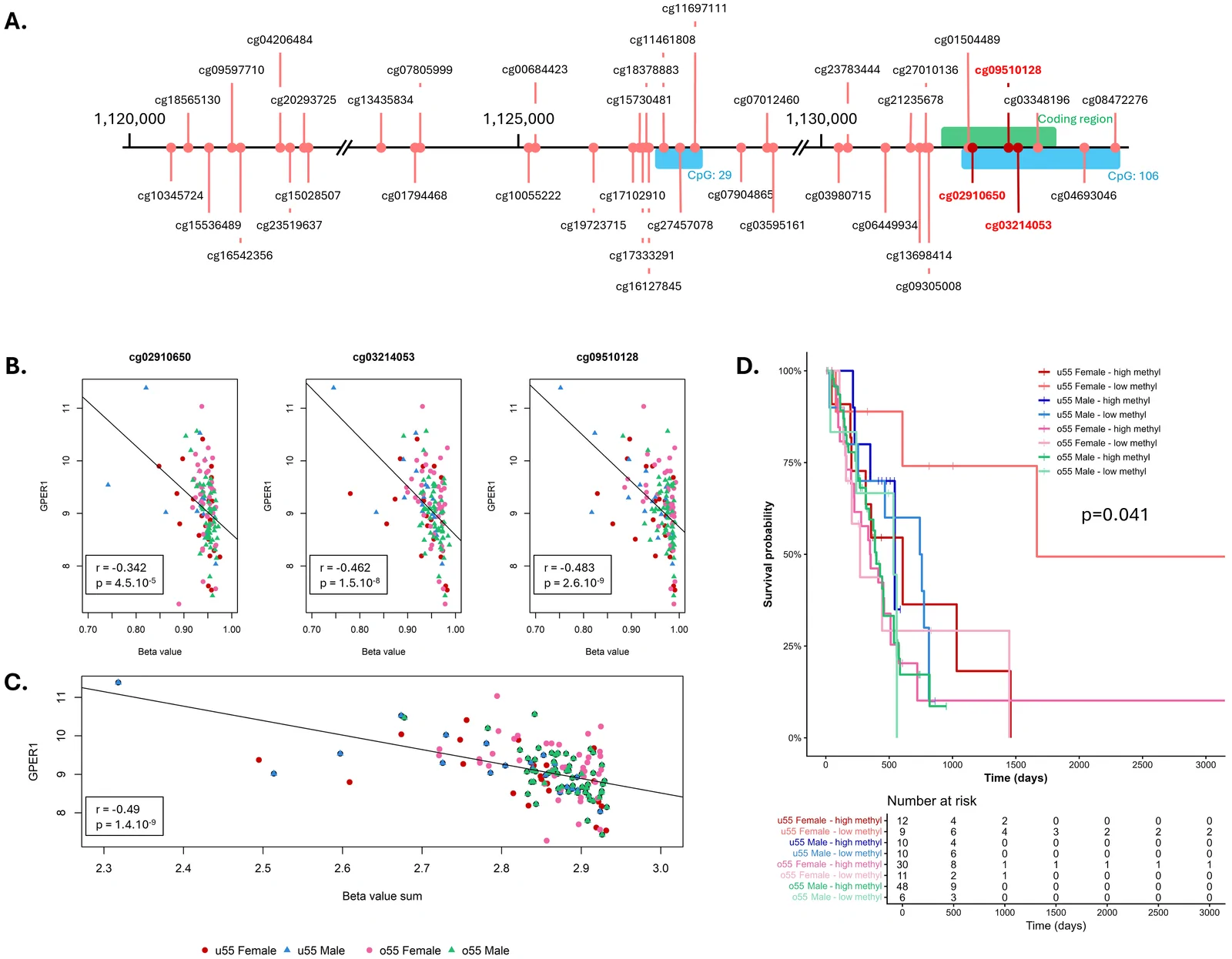
A low methylation of key CpG sites in *GPER1* locus is of favorable prognosis in u55 females. **(A)** Schematic representation of genomic structure of *GPER1* locus (located on chromosome 7p22.3) adapted from UCSC Genome Browser. The putative two CpG islands between positions chr7:1,126,579-1,126,910 and chr7:1,131,626-1,133,187 are indicated (blue) and contained 29 and 106 individual CpG sites respectively. The coding region between positions chr7:1,131,365-1,132,492 is indicated (green) (NCBI Reference sequence NC_000007.13). The analysis of methylation status was done using the Illumina 450K methylation data from reclassified TCGA-LGG and TCGA-GBM datasets according to WHO 2021 (n = 136). The data refers exclusively to the 40 CpGs within the *GPER1* locus for which methylation data were available for all patients. The cg identification numbers of the key 3 CpG sites which methylation is correlated with *GPER1* expression in GB are indicated in red. The correlation between the methylation level (Beta value) of each key CpG site **(B)** or the sum of Beta values for these 3 sites **(C)** and *GPER1* expression are reported according to patient sex and age. **(D)** The Kaplan-Meier plots were used to visualize the survival probabilities for the patients diagnosed with a GB according to WHO 2021. Kaplan-Meier estimates according to the combined methylation of the 3 keys CpG sites among patients’ sex and age.

## 4 Discussion

Sex hormones are increasingly recognized as key determinants of the sexual dimorphism observed in GB incidence, progression, and therapeutic response. Epidemiological data consistently show a lower incidence and improved survival in females, particularly before menopause (40). In the present study, we used a tailored workflow to select our cohort among male and female GB patients from both TCGA-LGG and TCGA-GBM datasets according to the WHO 2021 classification, since categorization of patients was initially based on the WHO 2008 and then the WHO 2016. A similar effort to reclassify histological tumor entities according to the most recent classification was published by De Mendonça et al. and led to reattribution of more than 50 samples (22). A Kaplan-Meier analysis confirmed the survival advantage of female aged less than 55 years old compared to other sex-and-age groups, supporting a protective role of active estrogen signaling in gliomagenesis. Future studies including direct hormonal measurements and detailed reproductive history would confirm the role of estrogen in the observed associations. Indeed, estrogen receptor signaling is increasingly recognized as regulating multiple hallmarks of glioma biology, including proliferation, invasion, stemness, and therapy resistance, through both tumor cell-intrinsic signaling and modulation of the tumor microenvironment via nuclear and membrane-bound hormone receptors (41, 42). ERβ expression decreases during glioma progression, while restoration or pharmacological activation of ERβ1 isoform suppresses proliferation, invasion, stem-cell maintenance, mesenchymal transition, and tumor growth in preclinical models. Mechanistically, ERβ1 inhibits oncogenic pathways including NF-κB, JAK/STAT3, PI3K/AKT, and TGF-β signaling, thereby reducing inflammatory and pro-survival transcriptional programs that are characteristic of aggressive GB phenotypes (16, 43). These observations are particularly relevant in light of recent evidence showing that NF-κB and STAT3 activation is associated with poor survival specifically in female GB patients, suggesting a potential interaction between estrogen signaling and sex-dependent inflammatory responses (44).

Furthermore, GPER has emerged as an important mediator of estrogen action in GB. High *GPER1* expression is associated with improved survival of IDHwt female patients treated by both radio- and chemotherapy (TCGA-GBM). Pharmacological activation of GPER using the selective agonist G-1 induces major anti-tumor effects, including mitotic arrest, *in vitro* and *in vivo* (17, 18). In previous study, high *GPER1* expression was associated with a longer survival in IDHwt females classified by WHO 2016 (TCGA-LGG) and *GPER1* correlated genes identified enrichment of genes involved in vasculature development such as *FLT4*, *ROBO4* and *TMEM204* (13). New data with combined TCGA-database allowed to obtain more IDHwt female patients (n=119) and to stratify them according to their age. We observed a survival advantage of u55 females, considered premenopausal, whose tumor expressed a high level of *GPER1*, even if *GPER1* expression average did not differ between sex and age. This suggested that the combination of a minimum expression threshold and the presence of a GPER activator, presumably estrogens present in the tumor macro-environment of young females, is required to trigger anti-tumoral signaling. Additional studies in cohorts for which estrogen exposure and circulating estrogen levels are precisely known for each patient will help refine the relationship between GPER expression/activity and survival prognosis in GB.

The integration of the two datasets enabled the identification of a larger repertoire of *GPER1*-correlated genes. In addition to previously reported genes, such as *FLT4*, *ROBO4*, and *TMEM204*, we identified novel genes whose association with *GPER1* appeared to be dependent on patient sex and age. Indeed, functional enrichment analysis of *GPER1* correlated genes in u55 females indicated that the specific signature of this group is characterized by 76 negatively correlated genes involved in cell cycle and genetic stability. This suggested that GPER can trigger a repressive signaling targeting mitosis/cell division. Interestingly, the transcriptomic profile of G-1 exposure consisted of genes whose expression was induced in response to treatment, yet all converged toward the same cellular effects, namely an alteration in the cell division process. Therefore, *GPER1* high expression and receptor activation could both trigger additive anti-proliferative effects through different pathways, ultimately leading to tumor growth arrest. Future studies using scRNA seq or computational cell-type deconvolution approaches would help determine the cellular origin of *GPER1* expression and further validate and strengthen our findings.

To highlight molecular mechanisms induced by GPER, initial studies relied on the use of agonists/antagonists. In GB, GPER agonist G-1 triggered anti-proliferative effects in U251 cells whereas Gutiérrez-Almeida et al. indicated opposite effect of G-1 in C6 rat GB cells (19). In cervical adenocarcinoma cells, G-1 modulates pathways involved in TNFα signaling, IL6/JAK/STAT signaling, oxidative metabolism, cell-cycle control, and apoptosis, supporting a global anti-proliferative program (45). However, the GPER-dependent effects of G-1 are still debated. In triple-negative breast cancer, Lv et al. showed that G-1 interacts directly with the colchicine binding site on microtubules to block cell division (20). Furthermore, Tutzauer et al. compared different receptor system assay to measure cellular response in the absence or presence of 17β-estradiol (E2) or G-1 and with or without GPER expression in several recombinant systems. GPER had no activity following E2 or G-1 treatments and can also have a ligand-independent activity (21).

Therefore, strategies aimed at investigating the effects of *GPER1* expression independently of agonist stimulation are relevant but remain poorly documented. In esophageal carcinoma, *GPER1* expression is frequently reduced, but its restoration suppresses tumor growth by activating the cAMP/PKA pathway, inducing ferroptosis, and ultimately enhancing apoptosis while reducing proliferation (46). Stable GPER overexpression decreases proliferation, migration, invasion, and cancer stem-like properties while promoting apoptosis in cervical adenocarcinoma cells. However, this tumor suppressive effect depends on the cellular context and histological entity (47). In cervical squamous cell carcinoma, GPER upregulated genes involved in tumorigenic pathways, including epithelial-to-mesenchymal transition (EMT), mTOR-C1, Myc, p53, hypoxia, and angiogenesis signaling. However, in cervical adenocarcinoma, these pathways are downregulated, confirming the divergent roles of GPER in cervical cancer cells (48).

In GB context, our study is the first to investigate the antitumor effect of GPER through transcriptomic analysis exclusively driven by its overexpression. Since individual hormone levels are not available in any TCGA dataset, we chose to culture U251 GB cells in DMEM medium without phenol red but containing fetal bovine serum, to represent the mean hormonal environment across all groups, rather than that of any particular group. Our experiments allowed us to draw conclusions regarding the effects of GPER overexpression specifically in tumor cells whatever their potential crosstalk with other cell types from a male or a female microenvironment. Thirty-four genes were identified (|FC| > 1.7, p < 0.05) regulated by GPER overexpression. None of these genes had been identified in Hambach’s studies, further supporting the hypothesis that the effects of GPER are dependent on the cellular context (48). Our data highlights a substantial proportion of long non-coding RNAs among genes regulated by GPER. This observation supports a potential role for GPER in regulating tumor cell plasticity through both coding and non-coding RNA-mediated mechanisms. Among them, GPER repressed the expression of *MALAT1* (Metastasis-Associated Lung Adenocarcinoma Transcript 1), a highly conserved lncRNA involved in the regulation of gene expression, alternative pre-mRNA splicing, cell proliferation, migration, and tumor progression (49). *MALAT1* expression was shown to be elevated in GB, and its knockdown inhibits proliferation, promotes apoptosis, and reduces invasion *in vitro*, reduces tumor volume and extends survival in an orthotopic GB murine model (50). Among the upregulated genes, *EGR1*, *CREB5* and *JUN* encode key regulators of gene expression and cellular processes, including cell proliferation. *EGR1* had also been reported to be upregulated in studies examining GPER impact via agonists such as G-1 (17, 51, 52), Bisphenol A (53), E2 and 4-hydroxitamoxifen (52, 54). *EGR1* was the only up regulated gene in common with our experiments and transcriptomic results following treatment with G-1 (17, 51), E2 and 4-hydroxytamoxifen (54), underscoring the relevance of our approach for identifying genes that are not induced by ligand stimulation and may instead be associated with ligand-independent GPER activity (17). EGR1 is a pleiotropic immediate-early transcription factor with context-dependent roles in GB, acting as either a tumor promoter or suppressor depending on the molecular environment. EGR1 is implicated in sex-specific oncogenic programs, cooperating with KLF5 and BRD4 to activate enhancer-driven transcription preferentially in male GB, where high expression correlates with poorer survival and increased tumor aggressiveness; its inhibition reduces growth and radioresistance in a sex-dependent manner (55). Conversely, several observations support a tumor-suppressive role for EGR1. EGR1 can transcriptionally activate PTEN and NAG-1, thereby inhibiting PI3K/AKT signaling, reducing invasion, and promoting apoptosis (56). Consistent with this mechanism, the PDE4 inhibitor Zl-n-91 suppresses GB growth through activation of the EGR1/PTEN/AKT signaling axis, leading to cell-cycle arrest and apoptosis both *in vitro* and *in vivo* (57).

Despite differences in the transcriptional targets identified following GPER overexpression and G-1 stimulation, comparable cellular phenotypes, notably anti-proliferative effects, were observed in U251 cells (17, 18). CMap analysis of their transcriptomic profile indicated that GPER overexpression signature is strongly associated with those of cell cycle disrupting compounds, like inhibitors of cell cycle progression, DNA transcription regulation and replication. Furthermore, Hirtz et al. indicated through the analysis of G-1 signature that many tubulin polymerization inhibitors had a connectivity score greater than 90 (17). All tubulin inhibitors associated with the G-1 signature were also found to be connected to *GPER1* overexpression, although SA-792574, podophyllotoxin, oxibendazole and chelidonine, were specifically related to *GPER1*. These observations suggest that pharmacological modulation of cellular pathways associated with GPER as well as alternative strategies aimed at restoring or enhancing *GPER1* expression deserve consideration, like the modulation of regulatory miRNAs. In GB, no fully validated microRNA network directly regulating *GPER1* has been established in GB. However, several GB-associated microRNAs, including miR-21, miR-155, and miR-124, are key regulators of major oncogenic signaling pathways such as PI3K/AKT and MAPK/ERK, suggesting that they may converge functionally on downstream *GPER1*-associated signaling rather than directly regulate *GPER1* itself (58–60).

Two CpG islands have been identified as key regulatory regions of *GPER1* expression. The first, designated as the downstream CpG island (dnCpGi), is located within the proximal promoter region and extends into the gene body, encompassing 106 CpG sites. The second is referred to as the upstream CpG island (upCpGi), located upstream of the transcription start site and contains 29 CpG sites. Within this upCpGi, Manjegowda et al. defined a differentially methylated region (DMR) of 8 CpG sites, whose methylation was associated with reduced *GPER1* expression (61). In colorectal cancer, where low *GPER1* expression is correlated with a poorer survival, it has been shown that methylation of several CpG sites (23 sites within a regulatory region overlapping with the upCpGi and 8 sites within the DMR) is inversely correlated with *GPER1* expression (62, 63). CpG methylation status was also assessed in TCGA breast cancer datasets. Manjegowda et al. identified a CpG site (cg11697111) located within the DMR whose methylation showed a significant inverse correlation with *GPER1* expression. In addition, they highlighted 4 CpG sites (cg15730481, cg17333291, cg07904865 and cg01719079) within the same regulatory region for which a composite methylation score exhibited an even stronger negative correlation with *GPER1* expression (61). In the present study, only CpG sites within the *GPER1* locus for which methylation data were available in all 136 IDH-wildtype patients from the combined TCGA-LGG and TCGA-GBM cohorts were analyzed. Among these sites, methylation levels at the 4 CpG loci previously highlighted by Manjegowda et al. (cg11697111, cg15730481, cg17333291, and cg07904865) did not show a significant association with *GPER1* expression. Nevertheless, we identified 3 CpG sites located within the dnCpGi whose methylation levels were significantly (p<0.05, |r|>0.3) and negatively correlated with *GPER1* expression. Notably, the inverse relationship between the combined methylation score of these 3 CpG sites and *GPER1* expression was consistently observed across all patient subgroups stratified by sex and age. Future validation in independent cohorts would strengthen our findings since the statistical power of the correlations decreases with successive stratification and reduced sample sizes (but remained 100% for male groups and at least 60% for female groups). However, the prognostic impact of these CpG sites appeared to be sex- and age-specific, as lower methylation levels were associated with improved overall survival only in females under 55 years of age. These findings suggest that CpG methylation may contribute to the regulation of *GPER1* expression and may influence the favorable prognosis observed in females classified as premenopausal. The association between lower methylation levels, higher *GPER1* expression, and improved survival supports the hypothesis that epigenetic re-expression of *GPER1* could represent a therapeutic opportunity in this specific patient subgroup. Indeed, in breast and colorectal cancer models, treatment with the DNA methyltransferase inhibitor 5-azacytidine has been shown to restore *GPER1* expression *in vitro* (61, 62). Further research is needed to assess whether modulation of *GPER1* locus methylation could have therapeutic relevance for the treatment of GB, particularly in u55 females.

## Data Availability

The datasets presented in this study can be found in online repositories. The names of the repository/repositories and accession number(s) can be found below: https://www.ncbi.nlm.nih.gov/geo/, GSE332587.
