# Supplementary material for: G-protein coupled estrogen receptor 1 (GPER1) expression improves survival of glioblastoma patients depending on sex and age: biological mechanisms and epigenetic regulation
Source: Front Endocrinol (Lausanne). 2026 Sep 25;17:1923928. doi: 10.3389/fendo.2026.1923928 (PMC13649380; doi:10.3389/fendo.2026.1923928)
Supplement: Supplementary file 1 [file DataSheet1.pdf]

Supplementary Table 1: Baseline survival and age characteristics according to patients' sex and age (n = 308).

	**Overall**	**Males**	**Females**
**All class of age**	308	189	119
Median age [years] [5%;95%]	60 [52;69]	60 [52;67]	60 [52;71.5]
Median survival [days]	370.5	382	357
**Over 55 years old (o55)**	207	126	81
Median age [years] [5%;95%]	65 [60;72]	65.5 [60.25;70]	65 [60;75]
Median survival [days]	318	326.5	279
**Under 55 years old (u55)**	101	63	38
Median age [years] [5%;95%]	48 [43;52]	50 [44;52]	47 [36.5;51]
Median survival [days]	442	544.6	534.5
