# Supplementary material for: G-protein coupled estrogen receptor 1 (GPER1) expression improves survival of glioblastoma patients depending on sex and age: biological mechanisms and epigenetic regulation
Source: Front Endocrinol (Lausanne). 2026 Sep 25;17:1923928. doi: 10.3389/fendo.2026.1923928 (PMC13649380; doi:10.3389/fendo.2026.1923928)
Supplement: Supplementary file 2 [file DataSheet2.pdf]

Supplementary Table 2: Genes significantly correlated with *GPER1* in each group (padj < 0.05). Negative correlations (r < −0.5) are shown in red, and positive correlations (r > 0.5) are shown in blue.

o55 males			u55 males			o55 females			u55 females		
gene	rho	padj	gene	rho	padj	gene	rho	padj	gene	rho	padj
PLXDC1	0.5629	1.38E-07	ROBO4	0.6861	1.10E-05	TBX2	0.6099	3.01E-05	ESAM	0.8319	1.95E-06
AGRN	0.5424	6.52E-07	DIPK2B	0.6755	1.68E-05	COX4I2	0.5827	1.15E-04	CLDN5	0.8039	1.63E-05
ROBO4	0.5398	6.52E-07	TMEM204	0.6489	8.90E-05	HIGD1B	0.5789	1.21E-04	HIGD1B	0.7658	1.70E-04
ESAM	0.5364	6.52E-07	FOXQ1	0.6413	1.20E-04	CLEC14A	0.5673	1.93E-04	CLEC14A	0.7597	2.13E-04
LAMC3	0.5362	6.52E-07	ESAM	0.6364	1.39E-04	JAG2	0.5672	1.93E-04	EDN1	0.7415	5.31E-04
DOCK6	0.5321	8.27E-07	AC007406.3	0.6296	1.76E-04	TMEM204	0.5604	2.63E-04	SRARP	0.7407	5.31E-04
FLT4	0.5273	1.11E-06	ARHGEF15	0.6275	1.76E-04	GPR4	0.5587	2.63E-04	APLN	0.7256	1.02E-03
ACE	0.5250	1.11E-06	CYSLTR2	0.6266	1.76E-04	PGAP6	0.5572	2.63E-04	EPAS1	0.7131	1.58E-03
FOXF2	0.5229	1.23E-06	FLT4	0.6224	2.07E-04	ESAM	0.5550	2.76E-04	DPEP1	0.7129	1.58E-03
SLC2A1	0.5194	1.42E-06	ACVRL1	0.6125	3.50E-04	MOCS1	0.5495	3.55E-04	TMEM204	0.7039	2.19E-03
DIPK2B	0.5186	1.42E-06	BCL6B	0.6093	3.89E-04	EXOC3L1	0.5485	3.55E-04	ROBO4	0.7011	2.38E-03
DAPK3	0.5120	2.39E-06	MYCT1	0.6007	5.79E-04	GJA4	0.5407	5.11E-04	DIPK2B	0.6976	2.67E-03
CDH5	0.5082	3.10E-06	LRRC32	0.6000	5.79E-04	NDUFA4L2	0.5385	5.11E-04	CDH5	0.6947	2.91E-03
SLC7A5	0.5062	3.47E-06	FOXF2	0.5983	6.00E-04	CLDN5	0.5384	5.11E-04	SLC3A2	0.6910	3.31E-03
TMEM204	0.5049	3.70E-06	GIPC3	0.5797	1.60E-03	ESRRA	0.5362	5.38E-04	TIE1	0.6818	4.66E-03
PFKL	0.5015	4.68E-06	PDGFB	0.5779	1.67E-03	FZD10	0.5352	5.38E-04	FLT4	0.6770	5.33E-03
ZBTB41	-0.5194	1.42E-06	HEYL	0.5764	1.71E-03	RASIP1	0.5349	5.38E-04	GRAP	0.6704	6.90E-03
BBIP1	-0.5261	1.11E-06	TBXA2R	0.5681	2.43E-03	ROBO4	0.5323	5.73E-04	NDUFA4L2	0.6652	8.05E-03
			RGS5	0.5678	2.43E-03	FOXS1	0.5264	6.90E-04	MYCT1	0.6588	9.75E-03
			NMUR1	0.5654	2.46E-03	GRIN2D	0.5222	7.93E-04	GNPTG	0.6579	9.75E-03
			RASIP1	0.5644	2.46E-03	STK25	0.5216	7.93E-04	BCAP31	0.6529	1.11E-02
			ACE	0.5642	2.46E-03	HRC	0.5141	1.06E-03	COQ9	0.6420	1.53E-02
			NOTCH4	0.5642	2.46E-03	PPP1R37	0.5119	1.11E-03	MYLK2	0.6411	1.53E-02
			FZD10	0.5626	2.49E-03	FLT4	0.5117	1.11E-03	SLC38A5	0.6391	1.57E-02
			GRAP	0.5619	2.49E-03	CORO1B	0.5116	1.11E-03	SLC2A1	0.6369	1.60E-02
			CDH5	0.5616	2.49E-03	SLC7A5	0.5079	1.27E-03	COX4I2	0.6350	1.62E-02
			PEAR1	0.5583	2.84E-03	PFKL	0.5070	1.28E-03	RGS5	0.6348	1.62E-02
			TNFRSF4	0.5577	2.84E-03	PTP4A3	0.5069	1.28E-03	NOTCH4	0.6339	1.62E-02
			MMRN2	0.5567	2.88E-03	LAMC3	0.5063	1.29E-03	SLC7A5	0.6323	1.68E-02
			AC026369.2	0.5479	4.14E-03	SOX18	0.5059	1.29E-03	B4GALT7	0.6317	1.69E-02
			GALNT18	0.5479	4.14E-03	GIPC3	0.5052	1.32E-03	RASIP1	0.6288	1.75E-02
			MMP25	0.5474	4.14E-03	ZNF503	0.5008	1.61E-03	C7orf50	0.6277	1.78E-02
			PCDH12	0.5470	4.14E-03	DENND1B	-0.5040	1.38E-03	LAMC3	0.6267	1.80E-02
			KANK3	0.5400	5.54E-03	ZNF644	-0.5082	1.27E-03	CSAG1	0.6251	1.82E-02
			APLN	0.5398	5.54E-03	RLIM	-0.5084	1.27E-03	PDGFB	0.6214	1.96E-02
			FCMR	0.5373	6.11E-03	DNAI4	-0.5139	1.06E-03	ECSCR	0.6164	2.22E-02
			ADGRL4	0.5355	6.46E-03	S100PBP	-0.5182	8.81E-04	MOB2	0.6162	2.22E-02
			HSPA12B	0.5335	6.90E-03	ZZZ3	-0.5184	8.81E-04	IGFBP1	0.6131	2.38E-02
			CLEC14A	0.5330	6.90E-03	RPAP2	-0.5200	8.46E-04	AC040169.1	0.6107	2.52E-02
			FOXL1	0.5308	7.47E-03	PMS1	-0.5228	7.93E-04	MOCS1	0.6067	2.81E-02
			SPAAR	0.5282	8.23E-03	AC026341.1	-0.5250	7.22E-04	C1QTNF1	0.6048	2.90E-02
			TM4SF18	0.5230	1.02E-02	DENND6A	-0.5268	6.90E-04	BCL6B	0.6030	3.05E-02
			MIXL1	0.5222	1.03E-02	THOC2	-0.5309	5.73E-04	EXOC3L1	0.5993	3.30E-02
			AP001122.1	0.5182	1.15E-02	C18orf25	-0.5311	5.73E-04	DCBLD1	0.5984	3.31E-02
			ADCY4	0.5182	1.15E-02	PIK3C3	-0.5322	5.73E-04	CHST12	0.5982	3.31E-02
			EXOC3L1	0.5178	1.15E-02	NSUN3	-0.5385	5.11E-04	ICAM2	0.5954	3.43E-02
			FGD5	0.5174	1.15E-02	CEP70	-0.5958	5.90E-05	GCAT	0.5930	3.58E-02
			EMCN	0.5168	1.15E-02				SLC6A12	0.5903	3.69E-02
			ANGPT2	0.5161	1.15E-02				MT3	0.5890	3.72E-02
			CSAG1	0.5156	1.16E-02				MMP28	0.5886	3.72E-02
			HCAR1	0.5120	1.33E-02				GYS1	0.5873	3.72E-02
			CD34	0.5113	1.35E-02				MT1M	0.5842	3.90E-02
			GPR4	0.5093	1.45E-02				MMP25	0.5829	4.01E-02
			DPEP1	0.5088	1.45E-02				AFAP1L1	0.5824	4.04E-02
			SLC7A5	0.5053	1.66E-02				LAMTOR4	0.5809	4.18E-02
			LAMC3	0.5012	1.95E-02				GGT5	0.5759	4.67E-02
			EXOSC2	-0.5164	1.15E-02				MT1X	0.5754	4.70E-02
			MRRF	-0.5212	1.06E-02				CCM2L	0.5730	4.94E-02
									EIF3K	0.5719	4.97E-02
									MADCAM1	0.5719	4.97E-02
									SMC2	-0.5722	4.97E-02
									LINC02511	-0.5726	4.97E-02
									ACVR2A	-0.5730	4.94E-02
									ZMYM1	-0.5730	4.94E-02
									FAM20B	-0.5735	4.94E-02
									TRIM33	-0.5761	4.67E-02
									ASNSP1	-0.5773	4.53E-02
									LINC01266	-0.5787	4.36E-02
									CAPN7	-0.5787	4.36E-02
									ETAA1	-0.5787	4.36E-02
									PMS1	-0.5803	4.24E-02

AC009533.2	-0.5816	4.12E-02
AC132154.1	-0.5831	4.01E-02
THUMPD1	-0.5851	3.82E-02
RIF1	-0.5862	3.72E-02
DNAJC10	-0.5864	3.72E-02
FKTN	-0.5866	3.72E-02
CCP110	-0.5868	3.72E-02
ERCC4	-0.5870	3.72E-02
EPM2AIP1	-0.5877	3.72E-02
MIS18BP1	-0.5879	3.72E-02
UBXN7	-0.5879	3.72E-02
AC027088.4	-0.5884	3.72E-02
AC009533.4	-0.5892	3.72E-02
NOL8	-0.5892	3.72E-02
RASA2	-0.5892	3.72E-02
PKN2	-0.5908	3.67E-02
MSH2	-0.5910	3.67E-02
ZBTB11	-0.5914	3.66E-02
AC026355.2	-0.5919	3.63E-02
ZNF281	-0.5923	3.63E-02
GOLGA4	-0.5940	3.49E-02
CEP170	-0.5943	3.49E-02
MSH6	-0.5951	3.43E-02
DCAF17	-0.5967	3.32E-02
SP3	-0.5969	3.32E-02
AC006299.1	-0.5972	3.32E-02
AP002371.1	-0.5977	3.31E-02
KIF20B	-0.5980	3.31E-02
CDIN1	-0.5991	3.30E-02
SMC6	-0.6008	3.18E-02
ZNF638	-0.6015	3.14E-02
SMURF2	-0.6024	3.08E-02
FANCL	-0.6050	2.90E-02
ZNF644	-0.6063	2.81E-02
PIAS1	-0.6083	2.69E-02
CCDC88A	-0.6105	2.52E-02
SMC5	-0.6105	2.52E-02
CCDC66	-0.6153	2.23E-02
CNTRL	-0.6157	2.22E-02
MRGPRF-AS1	-0.6172	2.20E-02
EMSY	-0.6179	2.17E-02
CREB1	-0.6197	2.07E-02
RPAP2	-0.6229	1.88E-02
AC009533.3	-0.6231	1.88E-02
CWC22	-0.6238	1.88E-02
LCORL	-0.6253	1.82E-02
NOP58	-0.6260	1.82E-02
ZNF654	-0.6275	1.78E-02
DENND6A	-0.6295	1.74E-02
C2CD5	-0.6306	1.70E-02
LPGAT1	-0.6306	1.70E-02
APPL1	-0.6339	1.62E-02
TMF1	-0.6343	1.62E-02
EIF2AK2	-0.6374	1.60E-02
SPOPL	-0.6383	1.59E-02
XPO1	-0.6404	1.53E-02
ZFP69B	-0.6404	1.53E-02
AP002812.2	-0.6423	1.53E-02
SENP1	-0.6464	1.38E-02
ZBTB41	-0.6490	1.27E-02
FBXO11	-0.6555	1.03E-02
AP000648.4	-0.6575	9.75E-03
SCN9A	-0.6634	8.38E-03
PRPF40A	-0.6658	8.05E-03
ZBTB6	-0.6787	5.14E-03
ARID4B	-0.6849	4.23E-03
CCDC18	-0.7103	1.69E-03
CEP57	-0.7129	1.58E-03
MRE11	-0.7361	6.20E-04
SMC4	-0.7693	1.67E-04
