# Supplementary material for: G-protein coupled estrogen receptor 1 (GPER1) expression improves survival of glioblastoma patients depending on sex and age: biological mechanisms and epigenetic regulation
Source: Front Endocrinol (Lausanne). 2026 Sep 25;17:1923928. doi: 10.3389/fendo.2026.1923928 (PMC13649380; doi:10.3389/fendo.2026.1923928)
Supplement: Supplementary file 3 [file DataSheet3.pdf]

Supplementary Table 3: List of genes specifically or commonly correlated with *GPER1* across the different groups, as identified by Venn diagram analysis.

o55 males	o55 males ∩ u55 males ∩ o55 females ∩ u55 females	o55 males ∩ u55 males	o55 males ∩ u55 females	o55 males ∩ u55 males ∩ u55 females	o55 males ∩ o55 females	u55 males	u55 males ∩ u55 females	u55 males ∩ o55 females	u55 males ∩ o55 females ∩ u55 females	o55 females	o55 females ∩ u55 females	u55 females
PLXDC1	ROBO4	ACE	SLC2A1	DIPK2B	PFKL	FOXQ1	BCL6B	GIPC3	RASIP1	TBX2	COX4I2	EDN1
AGRN	ESAM	FOXF2	ZBTB41	CDH5		AC007406.3	MYCT1	FZD10	CLEC14A	JAG2	HIGD1B	SRARP
DOCK6	LAMC3					ARHGEF15	PDGFB	GPR4	EXOC3L1	PGAP6	MOCS1	EPAS1
DAPK3	FLT4					CYSLTR2	RGS5			GJA4	NDUFA4L2	SLC3A2
	SLC7A5					ACVRL1	NOTCH4			ESRRA	CLDN5	TIE1
	TMEM204					LRRC32	GRAP			FOXS1	ZNF644	GNPTG
						HEYL	MMP25			GRIN2D	RPAP2	BCAP31
						TBXA2R	APLN			STK25	PMS1	COQ9
						NMUR1	CSAG1			HRC	DENND6A	MYLK2
						PEAR1	DPEP1			PPP1R37		SLC38A5
						TNFRSF4				CORO1B		B4GALT7
						MMRN2				PTP4A3		C7orf50
						AC026369.2				SOX18		ECSCR
						GALNT18				ZNF503		MOB2
						PCDH12				DENND1B		IGFBP1
						KANK3				RLIM		AC040169.1
						FCMR				DNAI4		C1QTNF1
						ADGRL4				S100PBP		DCBLD1
						HSPA12B				ZZZ3		CHST12
						FOXL1				AC026341.1		ICAM2
						SPAAR				THOC2		GCAT
						TM4SF18				C18orf25		SLC6A12
						MIXL1				PIK3C3		MT3
						AP001122.1				NSUN3		MMP28
						ADCY4				CEP70		GYS1
						FGD5						MT1M
						EMCN						AFAP1L1
						ANGPT2						LAMTOR4
						HCAR1						GGT5
						CD34						MT1X
						EXOSC2						CCM2L
						MRRF						EIF3K
												MADCAM1
												SMC2
												LINC02511
												ACVR2A
												ZMYM1
												FAM20B
												TRIM33
												ASNSP1
												LINC01266
												CAPN7
												ETAA1
												AC009533.2
												AC132154.1
												THUMPD1
												RIF1
												DNAJC10
												FKTN
												CCP110
												ERCC4
												EPM2AIP1
												MIS18BP1
												UBXN7
												AC027088.4
												AC009533.4
												NOL8
												RASA2
												PKN2
												MSH2
												ZBTB11
												AC026355.2
												ZNF281
												GOLGA4
												CEP170
												MSH6
												DCAF17
												SP3

AC006299.1
AP002371.1
KIF20B
CDIN1
SMC6
ZNF638
SMURF2
FANCL
PIAS1
CCDC88A
SMC5
CCDC66
CNTRL
MRGPRF-AS1
EMSY
CREB1
AC009533.3
CWC22
LCORL
NOP58
ZNF654
C2CD5
LPGAT1
APPL1
TMF1
EIF2AK2
SPOPL
XPO1
ZFP69B
AP002812.2
SENP1
FBXO11
AP000648.4
SCN9A
PRPF40A
ZBTB6
ARID4B
CCDC18
CEP57
MRE11
SMC4
