# Supplementary material for: G-protein coupled estrogen receptor 1 (GPER1) expression improves survival of glioblastoma patients depending on sex and age: biological mechanisms and epigenetic regulation
Source: Front Endocrinol (Lausanne). 2026 Sep 25;17:1923928. doi: 10.3389/fendo.2026.1923928 (PMC13649380; doi:10.3389/fendo.2026.1923928)
Supplement: Supplementary file 4 [file DataSheet4.pdf]

**Supplementary Table 4**: GPER overexpression target genes in U251 cells (|FC| > 1.7, $p < 0.05$).

| GeneSymbol | Regulation | Absolute FC | pval |
|---|---|---|---|
| ANKRD1 | up | 1.81 | 1.31E-03 |
| ARPC2 | up | 1.84 | 2.49E-05 |
| BRF1 | up | 2.25 | 1.13E-03 |
| CARD8 | up | 3.36 | 1.34E-06 |
| CHST13 | up | 1.85 | 2.79E-03 |
| CREB5 | up | 2.26 | 1.22E-03 |
| DHRS4 | down | 2.59 | 1.62E-06 |
| EGR1 | up | 4.39 | 3.70E-05 |
| GLI4 | down | 2.67 | 8.73E-05 |
| GPER1 | up | 11.21 | 3.02E-06 |
| HNF1B | up | 96.11 | 2.49E-09 |
| JUN | up | 1.82 | 2.36E-04 |
| KMT2E-AS1 | up | 2.24 | 4.85E-05 |
| LINC01088 | up | 3.46 | 1.88E-06 |
| LINC-PINT | up | 1.99 | 5.80E-04 |
| LMO7-AS1 | up | 5.57 | 1.01E-04 |
| lnc-LAT2-1 | up | 1.74 | 8.14E-03 |
| lnc-PLAU-2 | up | 2.05 | 1.94E-02 |
| lnc-RGS9-1 | down | 1.93 | 6.60E-05 |
| lnc-WRAP73-1 | down | 1.76 | 1.65E-04 |
| lnc-ZFHX4-2 | up | 1.90 | 4.73E-04 |
| LOC100129931 | up | 1.75 | 3.73E-03 |
| LRRN2 | up | 3.00 | 9.32E-03 |
| MALAT1 | down | 3.13 | 1.50E-05 |
| MIF | down | 1.80 | 4.17E-04 |
| NEAT1 | up | 2.89 | 1.51E-05 |
| NIP7 | down | 2.14 | 1.37E-05 |
| RHBDL1 | down | 1.71 | 6.18E-04 |
| SH2D2A | down | 8.32 | 1.19E-07 |
| SMIM38 | down | 36.11 | 1.80E-09 |
| SPOCK2 | up | 3.48 | 8.26E-07 |
| TACR2 | up | 26.62 | 3.23E-07 |
| TNFAIP3 | up | 1.84 | 9.26E-03 |
| YWHAE | up | 1.74 | 2.88E-03 |
